# Narrowing the rural oral healthcare gap: the 2017 rural interprofessional oral health practice symposium

**DOI:** 10.1186/s12903-018-0671-7

**Published:** 2018-12-20

**Authors:** Sean G. Boynes, Ce Ce Heyward, Christine Kenney, Abigail Lauer-Kelly, Joni Nelson, Kelli Ohrenberger, Christine Veschusio, Vuong Diep, Amy B. Martin

**Affiliations:** 1 The DentaQuest Institute, Westborough, MA 01581 USA; 2 Division of Population Oral Health, Department of Stomatology, James B. Edwards College of Dental Medicine; Medical University of South Carolina, Charleston, SC USA

Oral health disparities have been long established in rural America. Rural adults and children are more likely to report unmet need, unlikely to get involved in an oral health prevention program, and live in health provider shortage areas [1–8]. As the U.S. health care paradigm shift continues toward health as a goal, coordinated and integrated interprofessional care will become a key driver of health operations and the health care system’s financial structure. Oral health can serve as a link to chronic disease conditions that are amenable to team-based care, coordination across healthcare fields, and reallocation of resources from expensive treatment to more economical prevention. As successful and replicable oral health interprofessional practice (IPP) requires investment, ownership, and coordination, the DentaQuest Institute convened a Rural Oral Health Interprofessional Practice Symposium to build knowledge and ascertain areas of consensus that can help arch the interdisciplinary divide between dentistry and medicine. The goals of the symposium were to collect information and build knowledge around rural IPP as well as develop an understanding of: the current state of oral health care, what the future vision is in relation to a desired state, and what interprofessional action planning may close the gap between the two. This article provides an analysis overview of the discussions and findings of the 2017 Rural Interprofessional Oral Health Practice Symposium.

## Symposium process and methodology

The symposium was convened as a private event for 44 participant attendees over the course of two days in December of 2017. This group was comprised of leaders with different health backgrounds related to rural health care, oral health care, behavioral health, and health care policy. The attendees included representatives from State Offices of Rural Health; large healthcare systems; Schools of Medicine and Dentistry; dental service organizations, national and state Area Health Education Center Organization (AHEC), Primary Care Associations; Federally Qualified Health Centers; technology colleges; private practice; benefits companies; non-profit funders, coalitions and community based health organizations; and national healthcare organizations from 17 states. In addition, each participant identified their primary area of expertise that included: rural health (7); medicine (8); dentistry (10); public health (10); behavioral health (2); payer or funder (7).

Attendees were randomly assigned to a table number ranging from 1 to 6 based on the date of registration. Each table was assigned a table facilitator to serve as proctor for eliciting and recording responses to open discussions during three breakout sessions. Breakout sessions were organized according to a gap-analysis process: current state/what is happening now; desired state/vision for the future; and action planning to address the gap between current and desired states. Each breakout session was preceded by a three member expert panel that discussed the current landscape, the possible outcomes of healthcare’s paradigm shift to a more health oriented design, and the challenges and facilitators that can provide opportunity for interprofessional oral health practice. The Executive Director of the National Rural Health Association and Director of Interprofessional Practice of the DentaQuest Institute also provided a keynote presentation at the start of the convening as a means to provide appropriate background of rural interprofessional oral health practice. Poll Everywhere (San Francisco, CA), a text messaging application that allows participants to answer questions in real time, was employed throughout the symposium to solicit response to questions voluntarily submitted by attendees prior to the event. Table facilitators recorded and transcribed conversations for each table/small group. The transcribed conversations and Poll Everywhere responses were collected and collated for evaluation by an independent third party whom did not attend the symposium.

Presentations and discussions were organized into three sections:

- Current State of Oral Health Interprofessional Practice;
- The Vision or Desired State of Oral Health Interprofessional Practice; and
- Action Planning.

Content from the panel presentations and table discussions, as well as the Poll Everywhere results, were organized in these three sections.

## Symposium findings

### Section I: Current state of rural Oral health Interprofessional practice?

**Fig. 1:**
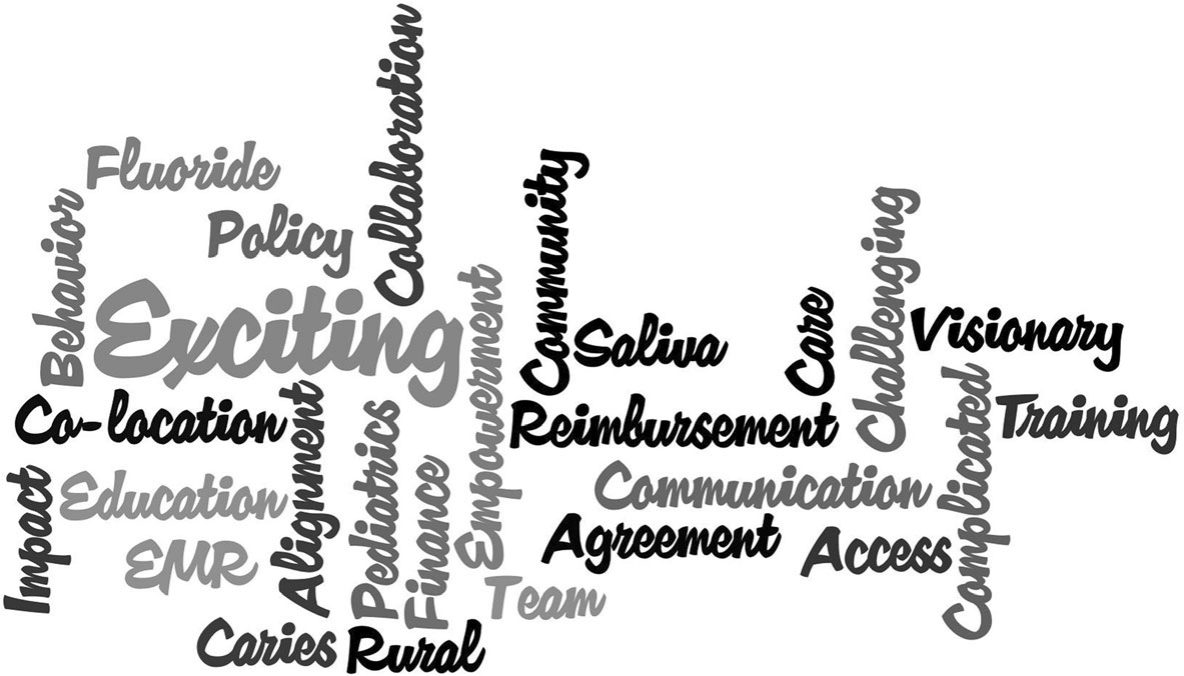
Word Cloud Responses from the Poll Everywhere Question: “In one word, describe the best opportunity to expand and enhance Interprofessional Oral Health Practice in the clinical setting”

**Fig. 2:**
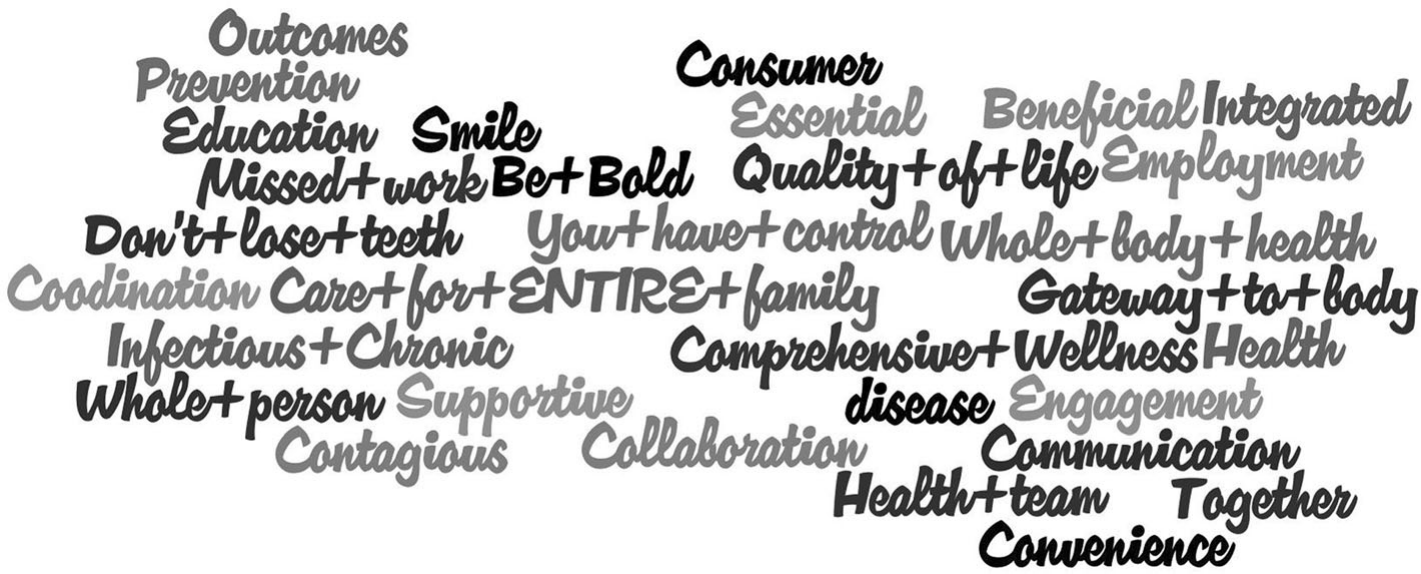
Word Cloud Responses from the Poll Everywhere Question: “In one to three words, describe the most beneficial primary public message/communication when it comes to rural oral health”

The first section opened with a Poll Everywhere survey. Responses are presented in Tables 1 and 2. The vast majority of attendees indicated a favorable impression of oral health interprofessional practice while acknowledging the barriers to its adoption, which included health information technology and workforce readiness. Attendees were also asked to identify one word that best describes the importance of interprofessional collaboration. Their responses are summarized in a word cloud in Figs. 1 and 2.

**Table 1:** Poll Everywhere Results for Section 1: What is Happening Now with Oral Health Interprofessional Practice

Question	Responses		
	Agree	Unsure	Disagree
Interprofessional oral health practice contributes to lower per capita cost; improve patient outcomes, and enhance the experience of care.	37	2	0
I have a growing frustration with the lack of timely progress with interprofessional practice (the integration and coordination of oral health care).	25	13	1
Health information technology systems and their vendors are currently a laggard that stagnates the proliferation of rural interprofessional oral health practice.	38^a^	1	0
The majority (50%) of providers in your state(s) of operation are entering the primary care setting are prepared to deliver the set of core competencies described by HRSA. (https://www.hrsa.gov/sites/default/files/hrsa/oralhealth/integrationoforalhealth.pdf)	1	12	25

^a^This was the only question that used a Likert scale response: Strongly agree (*n* = 17), Agree (*n* = 16), and Neutral (*n* = 5)

**Table 2:** Poll Everywhere Results for Section 1: The Vision/Desired State “Provide your agreement with the following statement: Value-based oral health care (incorporating interprofessional practice) will comprise 20% or more of the healthcare market”

Answer Choices	Responses
Within 5 years	8
Between 5 to 10 years	19
More than 10 years	11

The second activity of Day 1 was a panel presentation moderated by Christine Kavanagh, Nursing Faculty at Pennsylvania College of Technology and the following panelists were:

- Leah Elsmore, an Education Coordinator at Cavity Free at Three from the Colorado Department of Public Health and Environment
- Mary Ann Rigas, MD, a Pediatrician at the Cole Memorial Hospital System, Coudersport, Pennsylvania
- Christine Veschusio, DrPH, a Research Assistant Professor from the Division of Population Oral Health at the James B. Edwards College of Dental Medicine, Medical University of South Carolina in Charleston.

The panelists provided insights about the adoption of rural oral health interprofessional practice. Ms. Elsmore recalled from her feedback that 26% of her medical partners expressed concerns about promoting oral health during their patients’ primary care visits due to time constraints. Billing private insurance for fluoride varnish applications and general issues with their billing departments were identified by 12 and 11%, respectively. The number of partners was not provided.

Ms. Elsmore’s program, Cavity Free at Three, was working on a new pilot project at a federally qualified health center that focuses on providing oral health services to pregnant women. However, their challenges included (a) lack of communication between both departments and (b) collecting data on oral health services with limited coding unification. Ms. Elsmore gave the attendees two recommendations: focus on workforce development trainings for medical providers and promote oral health initiatives like Cavity Free at Three, in rural areas.

Dr. Rigas, a pediatrician from rural Pennsylvania, shared her challenges on adopting oral health interprofessional practices. As with Ms. Elsmore, getting private insurance to cover the costs of fluoride varnish was a challenge. Additionally, she identified the following challenges in her communities:

- patient recruitment into care,
- medical and dental provider shortages, particularly dentists willing to see children less than 3 years of age
- medical providers not trained in oral health
- families obtaining dental insurance
- families’ oral health literacy and low perceived values of dental care
- misperceptions on the safety of fluoridation
- socioeconomic factors such as poverty and lack of education
- Food quality and diet.

Dr. Rigas recommended attendees to consider taking several courses of action as rural communities seek to improve oral health interprofessional practice. She recommended oral health to be better incorporated into medical residency programs. For practicing clinicians, she also suggested pediatricians be paired with dental hygienists to foster interprofessional training in practice settings. Finally, she encouraged attendees to address how dentists can be included in the exchange of health information.

Through her experience, Dr. Veschusio provided an overview of a best practice in her home state of South Carolina. She and her team have been working with rural health clinics and dental offices to foster integrated care models that prioritizes adults with diabetes and children without dental homes. Using electronic health data in the practices, they were able to identify children in need of dental care. The rural health clinic staff then facilitated the establishment of a dental home. The program is called ROADS, Rural Oral Health Advancements in Delivery Systems. Through this experience, Dr. Veschusio acknowledged that electronic health records systems are nonexistent in the dental industry, which makes it difficult to rely on technology to facilitate referral partnerships. Additionally, she learned through ROADS that dentists do not fully understand their role in making referrals to physicians. Thus, they were unsure about how to develop formal referral partnerships with primary care providers.

Dr. Veschusio made several recommendations for attendees to consider as they think about collaborative referral partnerships between primary care and dentistry. One way to increase care capacity for Medicaid enrollees is to adopt the Safety Net Solutions business model, available through the DentaQuest Institute. Part of that adoption relates to how the dental practice can better collect and understand data, particularly in areas like tracking ‘no show’ rates. She acknowledged more research and data on the impact interprofessional practice has on patient outcomes are needed and that such analysis should be used to shape public policy.

At the conclusion of the panelists’ presentation, the moderator facilitated table discussions by asking three questions related to the current state of oral health interprofessional practice in rural communities. Each is subsequently explored.

Table Discussion Question #1: What are two best practices currently employed by innovators and early adopters in the current state of rural IPP practice?

Most of the discussions at the tables focused on recommendations for what participants would like to see, rather than current best practices. A fluoride varnish application by primary care providers was the most prevalent ‘best practice’ identified at the symposium with five tables of participants acknowledging it. Beyond its clinical impact, two tables discussed its effectiveness with establishing dental homes for patients and their families. Its weaknesses were also identified. One table discussed how it is an important tool but is inefficient for solving all a patient’s oral health problems. One participant confessed his or her practice does not get reimbursed for it because billing is a ‘time drain.’

The second best practice centered on new or emerging workforce models. Four tables discussed the role of dental hygienists, identifying them as leaders in primary care settings and possibly as the ideal team member for oral health-related case management. One table discussed the success of embedding a hygienist in primary care settings, particularly as a catalyst for integrated care. Two additional tables discussed community health workers as an emerging workforce in oral health interprofessional practice. The advantage of these team members is their presence in the community.

Table Discussion Question #2: What are the two main deficiencies in the system currently? Why are they deficient?

State policies and health information technology were identified as the two primary weaknesses for the adoption of oral health interprofessional practice. On the policy front, tables identified practice acts and reimbursement policies as the chief inhibitors. Four tables discussed their practice acts. One table specifically indicated the prior dental examination rule (that a dentist must complete a thorough intraoral examination prior to a non-dentist provide certain parameters of care) for hygienists to see in primary care settings as an obstacle to adoption. Four tables identified health information technology, specifically electronic health records as a difficulty with oral health interprofessional practice. The lack of clarity on what to chart and the poor functionality with referral management were discussed as deficiencies for both dental and medical care teams.

### Section II: The vision or desired state for rural Oral health Interprofessional practice

**Fig. 3:**
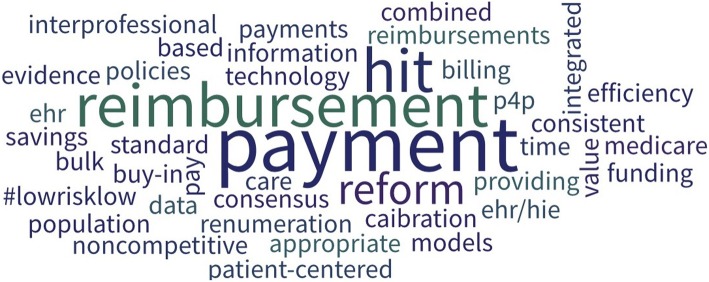
Word Cloud Responses from the Poll Everywhere Question: “In one word, describe the best opportunity to expand and enhance Interprofessional Oral Health Practice in the clinical setting”

Day 2 continued with a panel presentation on the vision or desired state of rural oral health interprofessional practice. As seen in Table 3 and Fig. 3, the second section was framed with two Poll Everywhere questions that included a word cloud response.

**Table 3:** Poll Everywhere Results for Section 2: The Vision/Desired State “Which of the following is the MOST important focus area that will result in positive return on investment for launching remote patient monitoring and treatment programs [teledentistry-tele-oral health]

Answer Choices	Responses
Cost savings financial business models	17
The creation, development, and proliferation of provider exchanges to provide dentists for supervision and advanced practitioners for mobile practice	5
A fee-for-service based systems that provides equal reimbursement for teledentistry	1
A pay scale for community based or outreach advanced practice practitioners (expanded hygienists / dental therapists) that is higher than office-based practitioners	4
Unanswered	17

The panel discussion prompted a conversation about the opportunities and challenges with the next step in IP oral health evolution. The panel discussed areas of active innovation and best practices that will be more prominent practice in the next 5–10 years. The moderator was Michelle Mills, Director of the Colorado State Office of Rural Health. Panelists were:

- Mark Deutchman, MD, Professor, Family Medicine, Associate Dean for Rural Health, School of Medicine, University of Colorado, Aurora, Colorado
- William Bailey, DDS, MPH, Endowed Chair in Prevention of Early Childhood Caries, School of Dental Medicine, University of Colorado, Aurora, Colorado and
- Alan Morgan, CEO for the National Rural Health Association.

The panelists discussed the opportunities and challenges with the next step in IPP and best practices anticipated within the next five to ten years. Dr. Deutchman indicated that higher education that confers IPP competencies and insurance payment reform were the two major priorities for advancing IPP. Dr. Deutchman discussed the paradigm with insurance coverage, more adults have medical insurance than dental insurance and because of this misalignment, the dental community has to rely heavily upon medical to address the importance of oral health for a comprehensive referral system to be established. Dr. Deutchman’s recommendations for best practices are:

- Broaden the view of oral health for dentists and start educating them on their role to other practitioners.
- Increase utilization of behaviorists to more adequately improve purposeful health behaviors.
- Medical professionals can help patients reduce anxiety experienced with dental visits.
- Since there are only 30 medical schools that offer rural health focused programs, we need to start finding more students with an interest in rural health and train them from there.

Dr. Bailey discussed innovation currently occurring with telehealth programs in Colorado, where he serves as an advisor. This is a virtual dental home based system that will provide services to patients who live 50 miles away utilizing private practice dentists in the community utilizing asynchronous and synchronous telehealth capabilities. Within this innovative model, dental hygienists visit assigned sites (school or workplace) to take radiographs and send to dentists for assessment and treatment planning. The importance of utilizing traditional private practice dental care teams to address access is vital to successful oral health. In addition, he stated that it is not advantageous to ignore the fact that financial sustainability and viability are necessary for any system change to occur and continue. Dr. Bailey had recommendations for best practices:

- Provide more IPP courses in alignment with accreditation standards.
- Federal agencies need to start cooperating with one another
- Promote telehealth in rural settings.

According to Mr. Morgan, there are a few ways to promote rural oral health (ROH) at the state and federal level. For example, policymakers can go to experienced sites and hear their stories on how they implement OH into their practice and utilize the media to gain their attention. He also denoted that the lack of coordination and collaboration at the Federal level and the lack of focus among stakeholders as threats that may hinder the promotion of ROH. The data on the efficacy of telehealth needs to be stronger, being that the previous datasets were from 2012, 2013 and 2014. At the completion of the panelists’ discussion, symposium attendees engaged in table conversations that addressed three questions relative to the vision or desired state of IPP.

Table Discussion Question #1: What are the two most important and one least important area of impact for the innovators and early adopters in the rural IPP practice in five years? Why are they least important?

The attendees were in agreement that technology was the most important, particularly telehealth technologies. Given its importance, concerns were expressed about rural communities’ abilities to support technology and the lack of health information technology interoperability. The second most important area of impact is the referral network. Having an effective and efficacious method for referral within rural is a key component. Least important was the use of mobile dentistry without comprehensive and continuous care. Tables agreed it to be inefficient, increases cost, and does not represent a dental home concept.

Table Discussion Question #2: In 2022, what do you think will be the main two deficiencies in the [rural interprofessional oral health] system?

The two main deficiencies expressed by attendees were finances and education, specifically the cost of obtaining and maintaining a teledentistry model in conjunction with concern about rural infrastructure capacity to support it. Financial policy out of alignment with practice was predicted for 2022 as well. Additionally, community support and education regarding IPP and teledentistry will increasingly become more important. In summary, the deficiencies identified in Section 1 [Current State] were validated by the panelists and predicted to remain threats in 2022.

Table Discussion Question #3: Is there one current state best practice that is superiorly positioned to transition to the future state?

One symposium table suggested participants look internationally, rather than domestically but did not offer specific successful models. The Mexican Dental Association was identified as having increased literacy. Lastly, the participants agreed the model presented by Dr. Bailey during the panel presentation represented the one best practice best positioned for future replication.

### Section III: Closing the gap: Action planning

Attendees were asked to provide resource recommendations for rural organizations and health systems working to integrate and coordinate oral health and explain why those resources are important. A few responses echoed findings from Section II [Ideal/Desired State] regarding the need and utilization of better communication methods between patients, dentists, and primary care providers. There is a need for more robust data analysis and evidence-based research to support the efficacy of better communication methods. Additionally, the implementation of technical assistance teams to assist practices with understanding oral health integration and operationalizing the available frameworks.

The attendees were also asked to provide a compelling argument for other health care providers to understand the value of oral health integration into primary care. The Smiles for Life Curriculum was offered as a way of bridging the gap for those providers who don’t understand or don’t see the value in integrated oral health care. Educating providers that oral health is connected to overall health and how it helps to prevent diseases is a major component.

The panel discussion within this section centered on closing the gap where they discussed opportunities and challenges regarding policy implications, challenges and the current state’s advantages will help with rural IPP. The panel was moderated by Brian Novy, DMD, Director of Practice Improvement at DentaQuest Institute and President of the DentaQuest Oral Health Center. Panelists were:

- Marcia Brand, PhD, MSD, Senior Dental Advisor, National Oral Health Programs, DentaQuest Foundation
- Carolyn Brown, DDS, President and Founder, Carolyn Brown and Associates, Inc.
- Anita Glicken, MSW, Executive Director of the National Interprofessional Initiative on Oral Health

Dr. Brand provided a few recommendations that are germane to creating an action plan for IPP, such as, sharing success stores with the champions who have had successful integration. Engaging with other federal systems, such as Veteran’s Affairs (VA) and Centers for Medicare and Medicaid Services (CMS) with the understanding that a lot of the populations served by these federal organizations are rural. She also reflected that guidelines for how federal agencies issue collaborative funding calls, or coordinate grants, with philanthropic organizations is unclear, however expressed confidence that federal agencies see rural oral health as a priority.

Dr. Brown stressed that an important piece in promoting IPP is standardizing the diagnosis coding system to ease transitions with and to value-based payment models. Dr. Brown’s recommendations for the action plan were to include more interprofessional education for dental students and use technology to promote oral health.

Ms. Glicken echoed Dr. Brown emphasizing the importance of interprofessional education and coupling education with HRSA competencies. She also mentioned establishing a common language between providers about what IPP means and how to accomplish as a component of the action plan. Ms. Glicken also noted that there are extremely limited metrics and data to support the known cost saving benefits of IPP, making the adoption difficult.

Dr. Novy directed a few questions to the symposium attendees:

Table Discussion Question #1: What impact area is the most important to assist with action planning to close the gap from current state to desired state?

The two most discussed impact areas were policy and communication and education for both the patient and the provider. As discussed by multiple panelists, the need for IPP education within medical and dental schools is an area of focus. The need for more robust data regarding cost savings and the interconnectivity of oral health and systemic health is important for those providers who are in practice and do not see IPP as a priority. Educating the patient is crucial to their health literacy and understanding. Policy changes are necessary to force a significant change in the way that practices provide care to their patients. Additionally, attendees discussed the importance of consumer buy-in and the need to enhance and improve the expectation that patients have of the care experience. Discussion on how to change consumer habits in a way that empowers patients to demand a care experience that manages health instead of disease occurred; however, pathways or solutions were not rectified within the table discussions.

Table Discussion Question #2: Name one high achieving goal to aid the oral health system transition to the 3.0 [next] era in healthcare.

Technology was noted as the most frequent response from the attendees. This was a major topic of the symposium and it was mentioned in all sections of the agenda. Technology and electronic health records are major issues and it needs to be comprehensively explored within the action planning phase.

Table Discussion Question #3: Name one low hanging goal to aid the oral health system transition to the 3.0 era in healthcare.

The most frequent response surrounded primary care integration with referrals, building a coalition and using community health workers for all relevant health issues. Empowering front desk staffs and nurses to promote oral health care within the primary care setting was another initiative for the action plan.

## Conclusion

Dissemination of the interprofessional practice concept has increased in the last two decades, most likely as the result of an ongoing paradigm shift that includes: consumer habit changes, focus on chronic disease management, politicization of U.S. Healthcare, increased provider dissatisfaction and burnout, and the healthcare cost crises [9]. This convening brought together stakeholders from or with an interest in rural health to develop and discuss the current and future state of interprofessional oral health practice in rural communities. The attendees described a currently fragmented system that would benefit from more consistency in the experience of care structure, utilization of payment or financial processes that direct health as a goal, and the design and agreement on outcomes based in population health. The attendees also recognized that many resources are available to assist care teams and organizations in integrating health systems; however, the operationalization of interprofessional practice as a tool has been slow and more technical assistance is needed to guide health systems toward truly integrated ventures. The convening concluded with participants focused on addressing inadequacies in educational standards and accreditation, an effort to change antiquated financial and practice act policy that impedes health care teams and systems from interprofessional practice, and involving more patients and consumers in decision making and education to change the expectation of oral health care.
